# Case Report: Neonatal Diabetes Mellitus Caused by a Novel *GLIS3* Mutation in Twins

**DOI:** 10.3389/fendo.2021.673755

**Published:** 2021-05-18

**Authors:** Shira London, Elisa De Franco, Ghadir Elias-Assad, Marie Noufi Barhoum, Clari Felszer, Marina Paniakov, Scott A. Weiner, Yardena Tenenbaum-Rakover

**Affiliations:** ^1^ Pediatric Endocrine Institute, Ha’Emek Medical Center, Afula, Israel; ^2^ Institute of Biomedical and Clinical Science, College of Medicine and Health, University of Exeter, Exeter, United Kingdom; ^3^ The Rappaport Faculty of Medicine, Technion – Institute of Technology, Haifa, Israel; ^4^ Clalit Health Services, Children Health Center, Naharia, Israel; ^5^ Faculty of Medicine, Bar-Ilan University, Zeffat, Israel; ^6^ Neonatal Intensive Care Unit, Ha’Emek Medical Center, Afula, Israel

**Keywords:** β-cell development, congenital glaucoma, congenital hypothyroidism, *GLIS3* mutation, neonatal diabetes

## Abstract

**Background:**

Mutations in *GLIS3* cause a rare syndrome characterized by neonatal diabetes mellitus (NDM), congenital hypothyroidism, congenital glaucoma and cystic kidneys. To date, 14 mutations in *GLIS3* have been reported, inherited in an autosomal recessive manner. GLIS3 is a key transcription factor involved in β-cell development, insulin expression, and development of the thyroid, eyes, liver and kidneys.

**Cases:**

We describe non-identical twins born to consanguineous parents presenting with NDM, congenital hypothyroidism, congenital glaucoma, hepatic cholestasis, cystic kidney and delayed psychomotor development. Sequence analysis of *GLIS3* identified a novel homozygous nonsense mutation, c.2392C>T, p.Gln798Ter (p.Q798*), which results in an early stop codon. The diabetes was treated with a continuous subcutaneous insulin infusion pump and continuous glucose monitoring. Fluctuating blood glucose and intermittent hypoglycemia were observed on follow-up.

**Conclusions:**

This report highlights the importance of early molecular diagnosis for appropriate management of NDM. We describe a novel nonsense mutation of *GLIS3* causing NDM, extend the phenotype, and discuss the challenges in clinical management. Our findings provide new areas for further investigation into the roles of GLIS3 in the pathophysiology of diabetes mellitus.

## Introduction

Neonatal diabetes mellitus (NDM) diagnosed before 6 months of age is a rare monogenic condition occurring in 1:100,000 live births in Europe (1, 2), and 1:30,000 (3-fold higher) in the Middle East (3, 4). The incidence of permanent NDM in the Middle East is even higher (10-fold) (1, 4). NDM accounts for 1:4000 cases of diabetes in children (5, 6). It may be either isolated or associated with multiorgan involvement. Genetic mutations causing NDM result in impaired insulin function due to one of the following mechanisms: abnormal pancreatic development, abnormal β-cell function or β-cell destruction. Mutations in over 25 genes have been reported to cause NDM (7–9), with syndromic NDM accounting for over 10% of the cases (9).

*GLIS3* encodes the zinc finger protein GLI-similar protein 3 (GLIS3), a transcription factor expressed in the developing pancreas and involved in the development of the thyroid, eyes, liver and kidneys (10–14). GLIS3 can function as a repressor or activator, playing critical roles in the regulation of various cellular processes (15, 16). In the pancreas, *GLIS3* is expressed at various stages of ductal and endocrine cell development, regulating β-cell development and insulin expression (12). Mutations in *GLIS*3 (OMIM 610192) were described in 2006 as the cause of a rare syndrome characterized by NDM, congenital hypothyroidism (CH) and congenital glaucoma (CG) (17, 18). Other features included intrauterine growth retardation (IUGR), cystic renal disease and hepatic cholestasis or fibrosis. In addition, developmental delay, facial dysmorphism, skeletal abnormalities and exocrine pancreatic dysfunction have been described (4, 17–22). To date, 14 *GLIS3* mutations have been reported (http://www.hgmd.cf.ac.uk/ac/gene.php) (4, 17, 20–22).

Herein, we report on two non-identical twins presenting with NDM, CG and CH caused by a novel homozygous *GLIS3* mutation. This case report expands our knowledge of the clinical phenotype, management approaches and outcome of infants with *GLIS3* mutations, indicating the need for additional research to further our understanding of the roles of GLIS3.

## Case Reports

### Case 1

**Figure 1:**
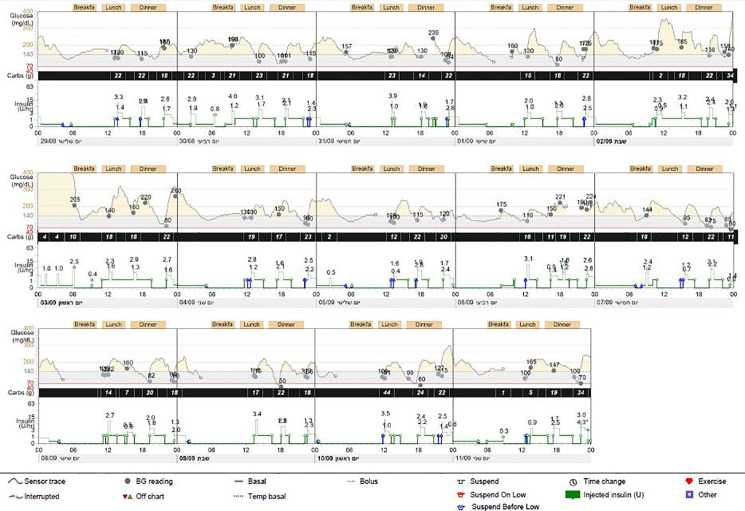
Pump report for case 1. Report from continuous glucose monitoring and from continuous subcutaneous insulin infusion (CSII) (MiniMed 640G) demonstrating several pump-disconnection episodes.

The proband was the firstborn male from an IVF dichorionic twin pregnancy born by spontaneous vaginal delivery at 37 weeks gestational age weighing 1984 g (-2.53 SDS) to healthy consanguineous parents (first cousins) of Arab-Muslim origin. No family history of diabetes was reported in the extended family. The newborn was admitted to the neonatal intensive care unit (NICU) due to low birth weight. In the first 24 h, he had low blood pressure that was treated with intravenous glucose 10%. On examination, no dysmorphic features were observed. Elevated blood glucose levels were observed during the first 24 h of life and through the first 5 days of life (range 61–443 mg/dL). The patient was treated with total parenteral nutrition for the first 4 days and was switched to milk formula at 5 days of age. He was treated intravenously with regular insulin from day 5 of life at a daily dose of 0.24-1.2 U/kg, which was replaced by a combination of subcutaneously injected diluted Humalog^®^ (insulin lispro) twice daily at a dose of 0.03–0.05 U before meals, and NPH insulin once or twice daily at a dose of 0.2–0.4 U/kg. Due to labile blood glucose levels, treatment with a continuous subcutaneous insulin infusion (CSII) pump (MiniMed 640G SmartGuard™ system) was initiated at 2 weeks of age. The infant weighed 1730 g. A MMT399 QuickSet Infusion Set was inserted into the upper lateral part of the buttocks. Regular insulin was administered at a basal rate of 0.3–0.6 U/kg per day with 2–3 boluses of 0.025 U each. The SmartGuard system offers protection against hypoglycemia by automatically stopping insulin instillation when the glucose sensor approaches a predefined lower limit. Maintaining stable blood glucose levels was very challenging due to fluctuating hyperglycemia and hypoglycemia (glucose levels ranged between 50 and 500 mg/dL). Hypoglycemic episodes were noted even after a few hours of insulin withdrawal, whereas low insulin-delivery rates resulted in pump obstruction, causing hyperglycemia. CSII using Humalog diluted 1:10 (10 U/mL) with continuous glucose monitoring (CGM) maintained blood glucose within 100–250 mg/dL, but the pump frequently shut down due to decreasing sensor glucose levels, on some days for more than 6 h (**Figure 1**). Laboratory assessment revealed very low levels of insulin (less than 0.2 µIU/mL; normal range 3.0–25.0), 0.1 ng/mL C-peptide (normal range 1.1–5.0) and negative anti-pancreatic antibodies (**Table 1**).

**Table 1:** Auxologic, biochemical and imaging findings in the twins.

		At presentation		Age 3 years		Normal values
		Twin 1	Twin 2	Twin 1	Twin 2	
Auxology	Weight (kg) (SDS)	1.98 (-2.53)	1.6 (-3.5)	14.35	16.6	
	Length (cm) (SDS)	ND	ND	98.0	98.6	
Biochemical results	Insulin (µIU/mL)	<0.2	<0.2	ND	ND	5-29.1
	C –peptide (ng/ml)	0.1	0.1	0.31	0.32	0.9-7.1
	Insulin Abs (%)*	Negative	Negative			<7.0
	GAD2 Abs (U/mL)*	Negative	7.2			<1.0
	IA2 Abs (U/mL)*	Negative	Negative			<0.75
	HbA1C (%)	8.2	7.4	6.5	7.0	<5.6
	TSH (mIU/L)	>150	>150	2.52	24.5	0.64-6.27
	FT4 (pmol/L)	<1.3	1.84	19.3	17.6	11.4-20.9
	FT3 (pmol/L)	1.99		8.1		3.5-6.5
Imaging	Abdominal US	ND	Normal	Normal	Normal	
	Kidney US with a 4 mm cyst.	Echogenic kidneys with small cortical cysts	Echogenic kidneys with small cortical cysts	Echogenic left kidney with a 4 mm cyst.	Normal	
	Thyroid US			Normal size and location	Normal size and location	
	Brain MRI			Normal	Normal	

ND, not done, US, ultrasound.

*Pancreatic antibodies were collected at 23 days.

Thyroid function testing during the first week of life revealed primary CH with thyroid-stimulating hormone (TSH) above 150 µIU/mL (normal range 0.64–6.27), free thyroxine (FT_4_) less than 1.3 pmol/L (normal range 11.4–19.0) and free triiodothyronine of 1.99 pmol/L (normal range 3.5–6.5). Thyroxine (L-T_4_) therapy was started at a dose of 15 µg/kg per day, which maintained thyroid function within the normal range after 2 weeks. During the follow-up period, his FT_4_ and TSH levels were within normal ranges or slightly elevated. Thyroid sonography demonstrated a normally sized non-ectopic thyroid gland.

Corneal opacity was noted early in the course of neonatal care and bilateral CG was diagnosed. Bilateral eye trabeculotomy was performed at 6 weeks of age. Repeat eye surgery was performed at 18 months of age due to increased intraocular pressure.

Renal sonography showed normally sized echogenic kidneys with small cortical cysts, mainly around the pyramids. Blood pressure and renal function tests were normal. Repeated renal sonography at the age of 3 years showed normally sized kidneys with small echogenic foci and small 4 mm cysts.

The baby developed transient jaundice with mildly elevated liver enzymes during the first days of life: glutamic oxaloacetic transaminase (GOT), 87 U/L (normal range 0–35); gamma-glutamyl transferase (GGT), 247 U/L (normal range 3–22); and alkaline phosphatase, 555 U/L (normal range 160–381). Total bilirubin was 6.26 mg/dL (normal range 0.3–1.2) with direct bilirubin of 0.69 mg/dL (normal range 0.1–0.3).The liver enzymes completely normalized on follow-up testing and abdominal ultrasonography was unremarkable, demonstrating normal size and texture of the liver, spleen, gallbladder and pancreas. Stool elastase was 396 µg/g (normal range >200), excluding exocrine pancreas insufficiency.

He was readmitted at 4 months and again at 9 months due to viral infection and pneumonia (respectively). During these hospitalizations, his insulin requirements increased 3- to 4-fold. At the age of 2.4 years, he was admitted to the ICU for prolonged severe hypoglycemia which occurred during an acute gastroenteritis episode (when he was off the insulin pump for 3 days). Hypoglycemia persisted despite three intramuscular injections of 1 mg glucagon administered by the parents and stabilized only after intravenous administration of glucose in the ICU. Adrenocorticotropin-stimulation test excluded adrenal insufficiency (basal cortisol of 2.09 µg/dL and peak cortisol at 60 min of 32.86 µg/dL; normal range >20).

At his last follow-up at the age of 3 years, his weight was 14.4 kg (40^th^ centile) and his height was 98 cm (60^th^ centile). His diabetes was well-managed (HbA1C was 6.5%) by CSII using Humalog diluted 1:10 (10 U/mL), with an insulin requirement of 0.7 unit/kg per day.

Auditory brainstem responses at 2 months of age revealed mild hearing impairment, but repeat examination at the age of 1 year was normal. Brain magnetic resonance imaging revealed no anomalies. He currently has mild global developmental delay and requires speech, physical and occupational therapy. He attends a kindergarten suited for children with special needs.

### Case 2

The proband’s twin sister was born weighing 1616 g (-3.5 SDS). Her birth, NICU hospitalization, and subsequent medical course were similar to that of her twin brother, with high blood glucose in the first 24 h (range 66–329 mg/dL). She was diagnosed with NDM, primary CH, and CG. Treatment with a CSII pump (MiniMed 640G SmartGuard system) was initiated at 16 days of age, when she weighed 1730 g. The management of her diabetes was similar to that of her brother’s with recurrent episodes of hypoglycemia even after a few hours of insulin withdrawal.

She had elevated GOT, 136 U/L, and total bilirubin was 8.04 mg/dL with 3.9 mg/dL direct bilirubin. Abdominal ultrasound was normal

Laboratory evaluation at presentation revealed low insulin and C-peptide. She had positive glutamic acid decarboxylase (GAD) antibodies of 7.2 U/mL (normal range <1) and negative anti-insulin and anti-islet cell antibodies at 23 days (**Table 1**). She was hospitalized at 4 months of age due to pneumonia, and again at 9 months due to a viral illness. During the hospitalizations, her insulin requirements increased 3- to 4-fold. Due to persistent tachypnea, she underwent echocardiography that revealed a normal heart. She was diagnosed with reactive airway disease and was treated with a corticosteroid inhaler (fluticasone propionate 125 µg twice daily). At her last follow-up at the age of 3 years, her weight was 16.6 kg (85^th^ centile) and height was 98.6 cm (75^th^ centile). Her diabetes was well managed (HbA1C of 7.0%) by CSII using Humalog diluted 1:10 (10 U/mL) with an insulin requirement of 0.9 unit/kg per day and with CGM. She currently has a mild to moderate delay in global development that requires speech, physical, and occupational therapy. She also attends a kindergarten suited for children with special needs.

### Molecular Analysis

The combination of NDM with CH, IUGR and CG raised suspicion of a mutation in *GLIS3*. Analysis of all coding regions and exon/intron boundaries of the *GLIS3* gene (transcript NM_001042413.1) was performed by PCR amplification (primer sequences available on request) followed by Sanger sequencing. Sequencing reactions were performed in an ABI3730 capillary machine (Applied Biosystems) and analyzed using Mutation Surveyor (SoftGenetics). A novel homozygous nonsense mutation located in exon 9, c.2392C>T, p.Gln798Ter (p.Q798*), was identified in both twins. The mutation introduces an early stop codon and is predicted to result in mRNA transcript degradation through nonsense-mediated decay. The genetic diagnosis was provided when the twins were 5 weeks of age. The parents are heterozygous carriers for the mutation. Another healthy sibling, born 1 year after the twins, was found through prenatal amniocentesis to be a heterozygous carrier of the same mutation.

## Discussion

We describe non-identical twins who are homozygous for a novel *GLIS3* mutation, presenting with NDM, CH, CG, kidney cysts and developmental delay. The management of their diabetes was challenging due to recurrent hypoglycemic episodes.

Since the first description in 2006 of *GLIS3* mutations causing a syndromic form of NDM in three siblings of a consanguineous family from Saudi Arabia (17, 18), an additional 16 patients have been reported carrying 14 different mutations (**Table 2**) (17, 19–23). All patients had NDM presenting between 1 and 31 days of life.

**Table 2:** Clinical and molecular findings of previous reported cases with *GLIS3* mutations.

No.	M/F	Consanguinity	Origin	B.W (kg)	NDM	Pancreas	Hypothyroidism	Eye	Kidney	Liver	Additional features	Mutation Type	References
1^a^	F	Yes	Saudi- Arabia	2.2	2 d	Small	Yes	No	Multiple small cysts	Fibrosis and canalicular cholestasis	Dysmorphism	c.2067insC (p.625fs703stop)	(17, 18)
2^a^	M	Yes	Saudi- Arabia	1.5	7 d	Not visualized	Yes	CG	Multiple small cysts	Fibrosis and canalicular cholestasis	Dysmorphism	c.2067insC (p.625fs703stop)	(17, 18)
3^a^	M	Yes	Saudi- Arabia	1.4	7 d	NA	Yes	CG	No	Hepatomegaly	Dysmorphism	c.2067insC (p.625fs703stop)	(17, 18)
4	F	Yes	Saudi- Arabia	1.64	Yes	Hypoplastic pancreas	Yes Agenesis	CG	No	Hepatomegaly	Dysmorphism	426kb-del/ 426kb-del	(17, 18)
5^b^	M	Yes	France	1.9	Yes	NA	Yes No uptake	No	No	No	Dysmorphism MR	149kb-del/ 149kb-del	(17, 18)
6^b^	M	Yes	France	1.8	Yes	Small	Yes Hypoplastic	No	No	Moderate steatosis	Dysmorphism MR	149kb-del/ 149kb-del	(17, 18)
7	F	Yes	Bangladeshi	1.17	3 d	NA	Yes (Normal) TSH resistance	No	Cystic dysplasia	Cirrhosis	Exocrine insufficiency, Osteopenia	Exons 1–2 del/ Exons 1-2 del	(20, 21)
8^c^	M	No	Welsh	1.43	4 d	Cystic change in the head of the pancreas	Yes (Normal) TSH resistance	No	Cystic dysplasia	Moderate parenchymal cholestasis	Exocrine insufficiency, Sensorineural deafness	Exons 1–4 del/ Exons 1-4 del	(20, 21)
9^c^	M	No	Welsh	2.02	2 d	NA	Yes	No	Renal cysts	Yes	Exocrine insufficiency	Exons 1-4 del/ Exons 1-4 del	(21)
10	F	No	Caucasian	2.75	2 d	NA	No	No	No	No	Choanal atresia	c.1765C>T p.Arg589Trp/Exons 1-11 del	(21)
11	F	Yes	Arab	1.75	2 d	NA	Yes (Agenesis)	No	Cystic dysplasia	Hepatitis	No	Exons 5–9 del/ Exons 5-9 del	(4, 21)
12	M	Yes	Arab	2.05	5 d	NA	Yes	No	No	No	Skeletal anomalies	c.1608C>G p.Cys536Trp/Cys593Trp	(4, 21)
13	F	NA	African-American	1.53	7 d	NA	Yes (Normal)	CG	Renal cysts	Cirrhosis	Sagittal craniosynostosis	Exons 9-11 del/ Exons 9-11 del	(21)
14	F	Yes	Yemeni	1.23	3 d	NA	Yes (Normal)	CG	Renal cysts	Hepatic fibrosis	Exocrine insufficiency	Exons 10-11 del/ Exons 10-11 del	(21)
15	F	Yes	Pakistani	1.86	1 d	NA	Yes	No	Renal cysts	No	Sensorineural deafness	c.932delG p.Gls311Alafs/ Gls311Alafs	(21)
16	M	Yes	Turkish	1.52	21 d	NA	Yes	No	Renal cysts	Hepatic fibrosis	No	Exons 3-4 del/ Exons 3-4 del	(21)
17	M	Yes	Kurdish	0.97	31 d	NA	Yes (Normal)	CG	Renal cysts	Hepatic fibrosis	PDA	c.1681C>T p.His561Tyr/ His561Try	(21)
18	M	Yes	Arab	1.7	19 d	NA	Yes	CG	Renal cysts	No	ASD	Exons 1-2 del/ Exons 1-2 del	(21)
19	M	Yes	Saudi-Arabia	1.3	2 d	NA	Yes	CG	No	No	No	c.2312_2314dupTC p.Pro722Leufs*35/ Pro722Leufs*35	(22)
20^d^	M	Yes	Arab	0.98	1 d	Normal	Yes	CG	Renal cysts	Hepatitis	Psychomotor delay	c.2392C>T p.Gln798Ter/ Gln798Ter	Current case
21^d^	F	Yes	Arab	1.6	1 d	Normal	Yes	CG	Renal cysts	Hepatitis	Psychomotor delay	c.2392C>T p.Gln798Ter/ Gln798Ter	Current case

^a,b,c,d^Siblings; CG, congenital glaucoma; BW, birth weight; NA, not available; ASD, atrial septal defect, PDA, patent ductus arteriosus; MR, mental retardation.

The molecular etiologies of NDM can be divided into three broad groups according to the defect that they cause in pancreatic function: abnormal β-cell function (*KCNJ11, ABCC8, INS, GCK, SLC2A2, SLC19A2*), β-cell destruction (dominant *INS, EIF2AK3, IER3IPI, FOXP3, WFS1, EIF2B1, LRBA*) and abnormal pancreatic development (*PDX1, PTF1A, HNF1B, RFX6, GATA4, GATA6, NEUROG3, NEUROD1, NKX2-2, MNX1, RFX6, CNOT1*) (6). The *GLIS3* gene is involved in the development of pancreatic β-cells and as such, belongs to the third group. In particular, *in-vivo* studies using *Glis3-*knockout mice have shown that Glis3 plays a critical role in cell-lineage differentiation, particularly in the development of β-cells, in the regulation of mature β-cell function and survival, and in insulin regulation (15).

In most patients with *GLIS3* mutations reported to date, and as was noted in our patients, a normal-size pancreas is observed. However, pancreatic hypoplasia and exocrine pancreatic insufficiency requiring pancreatic enzyme replacement and nutrient supplementation have been reported in some patients (21), but not in our cases. These findings support a possible role of GLIS3 in exocrine pancreatic development, as well as in β-cell development.

Patients with *GLIS3* mutations do not typically show any evidence of autoimmune markers, in contrast to type 1 diabetes mellitus in which the autoimmune destruction of β-cells is usually associated with the presence of pancreatic autoantibodies. Interestingly, anti-GAD antibodies were elevated in case 2. Although the presence of anti-GAD antibodies has been reported in rare cases of patients with non-autoimmune NDM (23), it would be interesting to investigate whether other patients with homozygous *GLIS3* mutations are positive for these antibodies. It would also be important to follow up our patient to determine whether she might revert to being GAD negative, as previously reported in some non-diabetic individuals (24). Of note, *GLIS3* gene polymorphisms have been associated with risk of type 1 diabetes mellitus in genome-wide association studies (25, 26), indicating that *GLIS3* is a susceptibility locus for this disease.

Both twins had intermittent episodes of hypoglycemia that resulted in insulin-pump disconnection. Moreover, in case 1, prolonged unresponsive hypoglycemia, resistant to glucagon therapy, was observed during an acute illness, requiring cessation of insulin therapy for 3 days. Other causes for hypoglycemia and adrenal insufficiency were excluded. Recurrent hypoglycemic episodes have been reported in one additional case with a *GLIS3* mutation (21). The mechanism through which these patients develop hypoglycemia is currently unknown, and further studies are needed to investigate its causes. Hypoglycemic episodes in the first days of life in small for gestational age infants are attributed to low liver glycogen storage. However, in our cases, the hypoglycemic events occurred during follow-up when the probands were older and at normal weight for their age. One explanation could be that GLIS3 dysfunction directly affects the regulation of insulin secretion. Indeed, GLIS3 expression persists beyond the embryonic period, promoting β-cell proliferation and β-cell survival, and regulating insulin gene (*INS*) expression by binding to GLIS3-response element in the *INS* promoter (16, 27). GLIS3 has been shown to activate pancreatic and duodenal homeobox 1 (*PDX1*), as well as neurogenic differentiation 1 (*NEUROD1*) expression and potently controls *INS* transcription (27). It has been shown that mice lacking *Glis3* have decreased numbers of glucagon-positive cells (28); however, the fact that no response to glucagon injections was observed in case 1 does not support low glucagon secretion as the cause for recurrent hypoglycemic episodes in these cases. Genome-wide association studies have revealed that *GLIS3* variants are associated with type 2, as well as type 1, diabetes mellitus (26), supporting its involvement in insulin sensitivity, which may explain the requirement of high insulin doses under stress conditions in our patients, as well as in other reports (20).

Our twins were homozygous for a novel nonsense mutation located in exon 9 (p.Q798*), which results in a premature stop codon. It is likely that the mutated transcript undergoes nonsense-mediated decay and little or no protein is produced. Five missense mutations of *GLIS3* have been reported to date; another eight mutations were intragenic deletions, and one was a duplication resulting in a frameshift (**Table 2**). A rapid molecular diagnosis of NDM is recommended as the specific etiology assists in clinical management (insulin *vs*. sulfonylurea), and may also guide longitudinal monitoring for other associated problems in NDM subtypes with syndromic features, as well as for screening family members (5, 9). Indeed, in the family reported on here, prenatal genetic testing revealed heterozygosity for the same *GLIS3* mutation in a later pregnancy.

Management of diabetes in our patients was challenging. The use of multiple insulin injections in infants results in labile blood glucose levels, and the use of NPH insulin commonly results in hypoglycemic episodes (5). Moreover, no insulin analogues are currently approved for use in infants (<1 year). CSII has been shown to provide the most effective management of NDM (5, 29, 30). However, insulin dilution is required to prevent pump obstruction. In contrast, the usefulness of CGM in neonates is arguable, since inaccuracy of CGM in newborns may lead to more interventions with potentially adverse effects on outcomes (31). The combination of CSII and CGM enabled the family to maintain blood glucose in a safe range and to prevent severe hypoglycemic events due to the automatic insulin-delivery shutoff system when the CGM predicts hypoglycemic values.

## Conclusions

We report a novel *GLIS3* mutation in non-identical twins, further characterizing the genotypic and phenotypic spectra in individuals with this rare genetic condition. Defining the clinical features of patients with rare forms of NDM is fundamental to providing insights into the roles of genes such as *GLIS3* in the pathophysiology of diabetes mellitus.

## Data Availability Statement

The raw data supporting the conclusions of this article will be made available by the authors, without undue reservation.

## Ethics Statement

The patients’ parents gave written informed consent for the publication of this case report.

## Author Contributions

SL, GE-A, MB, CF, MP, SW, and YT-R analyzed and interpreted the clinical and biochemical aspects of the patients’ data. EF analyzed and interpreted the molecular genetic data. SL, EF, SW, and YT-R contributed to writing the manuscript. All authors contributed to the article and approved the submitted version.

## Conflict of Interest

The authors declare that the research was conducted in the absence of any commercial or financial relationships that could be construed as a potential conflict of interest.
